# Perioperative statin medication impairs pulmonary outcome after abdomino-thoracic esophagectomy

**DOI:** 10.1186/s13741-022-00280-1

**Published:** 2022-09-14

**Authors:** Martin Reichert, Maike Lang, Joern Pons-Kühnemann, Michael Sander, Winfried Padberg, Andreas Hecker

**Affiliations:** 1 Department of General, Visceral, Thoracic, Transplant and Pediatric Surgery, University Hospital of Giessen, Rudolf-Buchheim-Strasse 7, 35390 Giessen, Germany; 2 Medical Statistics, Institute of Medical Informatics, Justus-Liebig-University of Giessen, Rudolf-Buchheim-Strasse 6, 35390 Giessen, Germany; 3 Department of Anesthesiology, Intensive Care Medicine and Pain Therapy, University Hospital of Giessen, Rudolf-Buchheim-Strasse 7, 35390 Giessen, Germany

**Keywords:** Esophagectomy, Esophageal cancer, Statin, Pneumonia, Ivor Lewis, Perioperative medication

## Abstract

**Background:**

Although surgery is the curative option of choice for patients with locally advanced esophageal cancer, morbidity, especially the rate of pulmonary complications, and consequently mortality of patients undergoing abdomino-thoracic esophagectomy remain unacceptably high. Causes for developing post-esophagectomy pulmonary complications are trauma to the lung and thoracic cavity as well as systemic inflammatory response. Statins are known to influence inflammatory pathways, but whether perioperative statin medication impacts on inflammatory response and pulmonary complication development after esophagectomy had not been investigated, yet.

**Methods:**

Retrospective analysis and propensity score matching of patients, who either received perioperative statin medication [statin( +)] or not [statin( −)], with regard to respiratory impairment (PaO_2_/FiO_2_ < 300 mmHg), pneumonia development, and inflammatory serum markers after abdomino-thoracic esophagectomy.

**Results:**

Seventy-eight patients who underwent abdomino-thoracic esophagectomy for cancer were included into propensity score pair-matched analysis [statin( +): *n* = 26 and statin( −): *n* = 52]. Although no differences were seen in postoperative inflammatory serum markers, C-reactive protein values correlated significantly with the development of pneumonia beyond postoperative day 3 in statin( −) patients. This effect was attenuated under statin medication. No difference was seen in cumulative incidences of respiratory impairment; however, significantly higher rate (65.4% versus 38.5%, *p* = 0.0317, OR 3.022, 95% CI 1.165–7.892) and higher cumulative incidence (*p* = 0.0468) of postoperative pneumonia were seen in statin( +) patients, resulting in slightly longer postoperative stay on intensive care unit (*p* = 0.0612) as well as significantly prolonged postoperative in-hospital stay (*p* = 0.0185).

**Conclusions:**

Development of pulmonary complications after abdomino-thoracic esophagectomy is multifactorial but frequent. The establishment of preventive measures into the perioperative clinical routine is mandatory for an improved patient outcome. Perioperative medication with statins might influence pneumonia development in the highly vulnerable lung after abdomino-thoracic esophagectomy. Perioperative interruption of statin medication might be beneficial in appropriate patients; however, further clinical trials and translational studies are needed to prove this hypothesis.

## Background

Esophageal cancer is one of the leading cancer diagnoses worldwide and esophageal resection is frequently decided as the curative option of choice (Pennathur et al. 2013). Thereby, esophageal cancer surgery is a high-risk procedure resulting in high postoperative complication and mortality rates being up to 60% and 14%, respectively (Zingg et al. 2011; McCulloch et al. 2003). Apart from anastomotic leakages, frequently observed postoperative complications are of pulmonary origin including respiratory distress and pneumonia (Reichert et al. 2020, 2019). The rates of postoperative pulmonary complications (PPC) range between 20 and 40%, and vice versa PPC majorly contribute to postoperative morbidity and mortality of the affected patients (Zingg et al. 2011; Avendano et al. 2002; Ferguson and Durkin 2002; Law et al. 2004; Blencowe et al. 2012; Seesing et al. 2018). Explanations for these high rates of PPC are vague. Perioperative atelectasis due to single-lung ventilation, postoperative pain after thoracotomy impairing the respiratory physiology, manipulation and injury of the thoracic cavity and the lung during surgery, and potential laryngeal nerve injury caused by extended lymph node dissection or cervical approaches to the esophagus resulting in an increased postoperative risk for aspiration were suggested to contribute to high PPC rates (Molena et al. 2014; Boshier et al. 2011; Bhayani et al. 2013). However, it has been observed that patients are significantly more vulnerable for impairments in pulmonary gas exchange and for development of PPC, especially pneumonia, after surgical approaches to the thoracic esophagus compared with major lung surgery, although both procedures result in a comparable operative trauma to the chest (Reichert et al. 2019).

Strategies to prevent postoperative pulmonary morbidity are almost lacking but are urgently needed. Hence, some authors advocate minimally invasive surgical approaches, especially to the abdominal cavity for gastric mobilization during abdomino-thoracic or abdomino-thoraco-cervical esophagectomy, resulting in improved postoperative pulmonary outcomes including lower pneumonia rates (Reichert et al. 2020; Mariette et al. 2019; Briez et al. 2012; Glatz et al. 2017). In the recent literature, this observation had been mainly attributed to lower abdominal pain levels after laparoscopic compared with open abdominal surgery (Mariette et al. 2019; Briez et al. 2012; Berlth et al. 2018; Sluis et al. 2019). In a previous work, we concluded that neither thoracotomy with surgical injury to the lung and to the thoracic cavity nor single-lung ventilation is mainly responsible for the high rates of respiratory impairment as well as PPC development after abdomino-thoracic esophagectomies (Reichert et al. 2019). However, a more indirect effect of trauma-induced systemic inflammation after (prolonged) conventional open abdominal surgery in comparison with hybrid minimally invasive, laparoscopically assisted esophagectomy might impair lung physiology and increases the risk to acquire pneumonia postoperatively (Reichert et al. 2020; D’Journo et al. 2010; Okamura et al. 2015; Katsuta et al. 1998; Babic et al. 2020). Considering this, some daily medication has the potential to influence inflammatory pathways and the immune reaction of surgical patients majorly, which, vice versa, may have an impact on postoperative patient outcome after major surgery. Statins, formally known as competitive inhibitors of the 3-hydroxy-3-methyl-glutaryl-CoA reductase, are a widely used every-day medication for hyperlipidemic and atherosclerotic patients, with a previously proven safety profile (Lemos et al. 2004). Beneath lipid-lowering and beneficial effects on atherosclerosis and subsequently on cardiovascular morbidity, statins are known to influence inflammatory pathways as recently reviewed by Koushki and colleagues (Koushki et al. 2020). Statins interfere with the expression of adhesion molecules and recruitment of inflammatory cells as well as decrease inflammasome activity and pro-inflammatory cytokine or acute phase protein production including interleukin (Il)-1, Il-6, tumor necrosis factor (TNF)-α, and C-reactive protein (CRP) (Koushki et al. 2020). These effects on inflammation are multifaceted and play a key role in plaque stabilization in atherosclerotic patients, but these effects may also play a role in other cases of systemic inflammation or infection (Koushki et al. 2020; Meyer et al. 2020; Krivoy et al. 2008; Meng et al. 2020; Jesus Oliveira et al. 2020; Oliveira et al. 2020). However, the influence of statins on perioperative inflammatory reaction and patient outcome, especially on postoperative pulmonary outcome in esophagectomy patients, has been poorly investigated until now. The present work aims to investigate the effect of perioperative statin medication on postoperative respiratory dysfunction as well as pneumonia rates following abdomino-thoracic esophagectomy.

## Methods

### Patients

This retrospective single-center cohort study was formally approved by the local ethics committee of the medical faculty of the University of Giessen (approval no. 214/15 and 253/16). The study was performed in accordance with the latest version of the Declaration of Helsinki. The data are collected and the manuscript is written and submitted in accordance with the COPE guidelines. All patients were treated according to the institutional standard of care.

From 01/2009 to 12/2017, all consecutive patients who underwent abomino-thoracic (Ivor Lewis) or abdomino-thoraco-cervical (McKeown) esophagectomy for cancer were included in the data analysis. Patients, who underwent a transhiatal surgical approach to the esophagus or who underwent re-do surgery for local recurrent esophageal carcinoma, were excluded from the study. Further exclusion criteria were multivisceral abdominal surgery with pancreatic resection for locally advanced carcinoma of the esophago-gastric junction, benign disease or esophageal perforation necessitating esophageal resection, abdomino-cervical esophagectomy without a trans-thoracic part of the procedure, and cervical esophagectomy for carcinoma of the larynx or hypopharynx.

Patient data were analyzed retrospectively from the prospectively maintained institutional database regarding general patient characteristics and surgical procedure characteristics, general postoperative patient outcome and more specifically regarding perioperative blood leukocyte counts and C-reactive protein (CRP) values from routine laboratory examinations, postoperative pulmonary outcome including duration of invasive mechanical ventilation through endotracheal tube or tracheotomy, re-intubation and tracheotomy rates, postoperative pneumonia, and perioperative oxygenation indices. The patient cohort was divided with regard to perioperative statin medication, which was obtained from medical records.

The rate of pneumonia was assessed retrospectively until postoperative day 30 (POD 1–30) after abdomino-thoracic or abdomino-thoraco-cervical esophagectomy. Therefore, the “Revised Uniform Pneumonia Score,” which had been previously validated for patients after esophagectomy, was used (Weijs et al. 2016). The postoperative day of retrospective pneumonia diagnosis was recorded for pairwise comparisons as well as cumulative incidence calculation. As described previously, the scoring system was minimally modified: we decided a body temperature ≥ 38.0 °C or ≤ 36.0 °C, respectively, as the threshold for pneumonia scoring in the present study according to the current “International Guidelines for the Management of Severe Sepsis and Septic Shock 2012” (Reichert et al. 2020, 2019; Weijs et al. 2016; Hecker et al. 2019).

The oxygenation index (i.e., PaO_2_/FiO_2_ ratio or P/FR) is a well-known parameter for the evaluation of pulmonary function. P/FR values were regularly available for patients staying in the intensive care unit (ICU) and adequate respiratory function is an important general clinical criterion for discharge of patients from the ICU to the normal ward after major surgery. Thus, discharge from the ICU was consequently interpreted as the absence of respiratory impairment (or failure) in this study and a “normal oxygenation” with a P/FR ≥ 300 mm Hg was anticipated for patients on the normal ward. In the present work, P/FR was calculated as the ratio of the arterial pressure of oxygen (PaO_2_) and the fraction of inspired oxygen (FiO_2_) (PaO_2_/FiO_2_) as described previously (Ranieri et al. 2012) at different time points: at the beginning of mechanical ventilation (under double-lung ventilation), upon arrival at the ICU (POD 0), and on POD 1–10 (Reichert et al. 2020, 2019). If the PaO_2_ and FiO_2_ were measured more than once a day, the first values of the day were used. According to the Berlin classification, a P/FR ≤ 300 mmHg is an important criterion for the clinical definition of acute respiratory distress syndrome (Ranieri et al. 2012); thus, a P/FR < 300 mmHg was considered to indicate respiratory impairment or failure (Reichert et al. 2020, 2019). For mechanically ventilated patients (either invasively or non-invasively), the FiO_2_ was available. For patients, who were not mechanically ventilated (invasively or non-invasively) in the ICU, a FiO_2_ of 30% was assumed to globally take nasal oxygen supply into consideration in these patients immediately following extubation. To focus on acute respiratory insufficiency making re-intubation necessary, we assessed postoperative re-intubations independently from re-do surgery.

### Surgery and perioperative patient care

Two-incision, abomino-thoracic esophagectomy (Ivor Lewis) for lower- or mid-esophageal malignoma or cancer of the esophago-gastric junction or three-incision, abdomino-thoraco-cervical (McKeown) esophagectomy for higher located esophageal malignoma are widely used standard surgical procedures (Pennathur et al. 2013). The institutional technique was described previously (Reichert et al. 2020, 2019). All patients included in this study underwent right-sided, anterolateral thoracotomy for the thoracic part and either laparotomy or laparoscopy for the abdominal part of the esophagectomy procedure. Patients who underwent conversion from an initially intended hybrid, minimally invasive, laparoscopically assisted approach to laparotomy were attributed to conventional open surgery.

All patients underwent either a two-field or a three-field lymph node dissection dependent on the extent of surgery (abdomino-thoracic or abdomino-thoraco-cervical esophagectomy) following international recommendations (Pennathur et al. 2013). All abdomino-thoracic or abdomino-thoraco-cervical esophagectomy procedures reported in this study were performed in one surgical intervention. In most cases, the thoracic part followed the abdominal part of the surgery. Reasons for a thorax-first approach were the determination of local resectability. The (subtotal) esophagectomy and reconstruction of the esophago-gastric continuity were regularly completed trans-thoracally by gastric pull-up. In one case included in this study, esophago-gastrectomy was performed. In this case, the continuity was restored by colonic interposition.

The duration of the thoracic part of the surgical procedure was calculated by the duration of single-lung ventilation or the time of the thoracic incision upon retrospective availability of data, respectively.

Postoperatively, patients were treated by principles of a “fast track” protocol including early extubation, early enteral nutrition, and early mobilization (Rubinkiewicz et al. 2019; Low et al. 2019; Moorthy and Halliday 2021). Patients were monitored at the ICU for at least until POD 1 and—if cardiac and respiratory functions were stable—discharged from the ICU.

### Statistical analyses

The patient cohort was divided into either group of patients who received a statin medication perioperatively [statin( +)] or who did not [statin-naïve or statin(–)].

Statistical analyses were performed using GraphPad Prism (version 5.00 for Windows, GraphPad Software, San Diego, CA, USA, www.graphpad.com). Categorical data of both groups were analyzed using Fisher’s exact or Pearson’s *X*^2^ test. Mann–Whitney *U* test was used to perform two-group comparisons of continuous variables. Patients who died were excluded from the analysis upon the day of death.

Odds ratios (OR) and 95% confidence intervals (95% CI) were used to calculate the effect size of perioperative statin medication on postoperative pneumonia development by the Baptista-Pike method.

Cumulative incidences of postoperative pneumonia during POD 1–30 and postoperative respiratory impairment (defined by a P/FR < 300 mmHg) during POD 1–10 of statin-naïve and statin( +) patients were calculated by Kaplan–Meier estimation, as described previously (Reichert et al. 2020, 2019). P/FR on POD 0 (arrival on ICU) was not included in the cumulative incidence calculation. Kaplan–Meier curve comparisons were performed by log-rank test. Patients who were discharged, died, or underwent re-do surgery were censored from the analysis of cumulative incidences. Vertical ticks in the figures indicate censored data.

To obtain a better comparability of both groups regarding patient characteristics, 1:2 [statin( +) patients: statin( −) patients] propensity score pair matching (PSM) with a match tolerance = 0.0 was performed by the package “MatchIt” with “R” (version 4.0.3) followed by a repeat of the analyses as described above. The propensity score of the patients was calculated including relevant variables of preoperative patient characteristics and relevant characteristics of the surgical procedure [American Society of Anesthesiologist’s classification of physical health (ASA) score, chronic pulmonary diseases, Charlson Comorbidity Index (CCI) and conventional open or hybrid minimally invasive, laparoscopically assisted surgery].

Finally, to determine statistical dependences between perioperative statin medication and postoperative pneumonia as well as postoperative pneumonia and postoperative leukocyte counts and C-reactive protein values, Spearman’s rho rank correlation was used. Results are given as Spearman’s rank correlation (*r*_sp_) and respective significances.

Data are given in tables as medians with minimum to maximum ranges for continuous variables as well as *n* (%) for categorical variables; *p*-values ≤ 0.05 indicate statistical significance. Because of the exploratory character of the study, no adjustments of *p*-values were performed.

## Results

### General characteristics of the unmatched patient cohort

Between 2009 and 2017, 117 patients, who underwent Ivor Lewis (abdomino-thoracic) or McKeown (abdomino-thoraco-cervical) esophagectomy for oncologic purposes, were included into the data analysis. The patient cohort was subdivided into the group of patients who received a statin medication perioperatively [statin( +): *n* = 26, 22.2%] or not [statin( −): *n* = 91, 77.8%]. Patient characteristics of both native, unmatched groups are shown in Table 1. Overall, 87 (95.6%) statin-naïve patients and all patients from the statin( +) group preoperatively had evidence of chronic diseases (*p* = 0.5739). Of note, patients from the statin( +) group were at baseline significantly older and suffered from more severe chronic diseases including arterial hypertension, coronary artery diseases, and (by tendency) chronic lung diseases, which is reflected by higher ranks in the ASA score. Higher rates of co-morbidities in the characteristics of patients from the statin( +) group and vice versa might cause higher rates of primary surgery without previous induction therapies, although these patients had evidence for locally more advanced histo-pathological tumor stages (*p* = 0.0084), accordingly for locally more extended esophageal surgery [McKeown procedure with cervical anastomosis was performed in 3.3% of statin( −) and 23.7% of statin ( +) patients, *p* = 0.0037] and these patients were approached by tendency less frequently by a hybrid minimally invasive, laparoscopically assisted approach (*p* = 0.0758, Tables 1 and 2). However, no differences were observed in additional procedures to the esophagectomy, nor in the duration of surgery and intraoperative blood loss (Table 2).

**Table 1:** Patient characteristics

Variable	All patients			Propensity score-matched patients		
	Statin ( −) patients (*n* = 91)	Statin ( +) patients (*n* = 26)	*p* value	psmStatin ( −) patients (*n* = 52)	psmStatin ( +) patients (*n* = 26)	*p* value
Type of statin [*n*]	-		-	-		-
Lipophilic						
Simvastatin		19 (73.1%)			19 (73.1%)	
Atorvastatin		4 (15.4%)			4 (15.4%)	
Hydrophilic						
Pravastatin		3 (11.5%)			3 (11.5%)	
Male gender [*n*]	72 (79.1%)	23 (88.5%)	0.3970	39 (75.0%)	23 (88.5%)	0.2371
Age [years]	64.0 (40.0–85.0)	67.5 (53.0–86.0)	0.0224	63.5 (40.0–85.0)	67.5 (53.0–86.0)	0.0536
BMI [kg/m^2^]	24.2 (15.6–41.3)	25.0 (20.3–37.6)	0.0841	24.1 (16.0–41.3)	25.0 (20.3–37.6)	0.1407
ASA [median]	2 (1–4)	3 (2–4)	< 0.0001	3 (2–4)	3 (2–4)	0.2336
1 [*n*]	3 (3.3%)	0		0	0	
2 [*n*]	47 (51.6%)	3 (11.5%)		11 (21.2%)	3 (11.5%)	
3 [*n*]	39 (42.9%)	21 (80.8%)		39 (75.0%)	21 (80.8%)	
4 [*n*]	2 (2.2%)	2 (7.7%)		2 (3.8%)	2 (7.7%)	
History of malignancy [*n*]	14 (15.4%)	6 (23.1%)	0.3817	10 (19.2%)	6 (23.1%)	0.7690
Abuse [*n*]						
Alcohol	19 (20.9%)	5 (19.2%)	1	11 (21.2%)	5 (19.2%)	1
Smoking	33 (36.3%)	12 (46.2%)	0.3709	18 (34.6%)	12 (46.2%)	0.3365
Arterial hypertension [*n*]	50 (54.9%)	22 (84.6%)	0.0062	34 (65.4%)	22 (84.6%)	0.1093
Coronary artery disease [*n*]	10 (11.0%)	10 (38.5%)	0.0024	9 (17.3%)	10 (38.5%)	0.0524
Chronic lung disease [*n*]	17 (20.9%)	10 (38.5%)	0.0617	12 (23.1%)	10 (38.5%)	0.1867
Chronic kidney failure [*n*]	6 (6.6%)	4 (15.4%)	0.2259	5 (9.6%)	4 (15.4%)	0.4713
Previous abdominal surgery [*n*]	28 (30.8%)	5 (19.2%)	0.3262	12 (23.1%)	5 (19.2%)	0.7783
Induction therapy [*n*]	63 (69.2%)	11 (42.3%)	0.0199	36 (69.2%)	11 (42.3%)	0.0285
Indication [*n*]						
Malignancy			0.9162			1
Adenocarcinoma	56 (61.5%)	15 (57.7%)		30 (57.7%)	15 (57.7%)	
SCC	31 (34.1%)	10 (38.5%)		20 (38.5%)	10 (38.5%)	
Others	4^a^ (4.4%)	1^b^ (3.8%)		2^c^ (3.8%)	1^b^ (3.8%)	
Pathological tumor stage^d^						
T 0	19^e^ (21.6%)	0	0.0084	11^e^ (21.6%)	0	0.0428
T 1	9 (10.2%)	4 (16.0%)		7 (13.7%)	4 (16.0%)	
T 2	23 (26.1%)	9 (36.0%)		15 (29.4%)	9 (36.0%)	
T 3	37 (42.1%)	10 (40.0%)		18 (35.3%)	10 (40.0%)	
T 4	0	2 (8.0%)		0	2 (8.0%)	
N 0	53 (58.2%)	13 (50.0%)	0.5055	30 (57.7%)	13 (50.0%)	0.6305
N +	38 (41.8%)	13 (50.0%)		22 (42.3%)	13 (50.0%)	
M + ^f^	5 (5.5%)	1 (3.8%)	1	4 (7.7%)	1 (3.8%)	0.6598

Data are given in median and minimum to maximum ranges or *n* (%).^a^Including one salvage esophagectomy after primary radio-chemo-therapy as well as sarcoma, neuroendocrine carcinoma, and gastrointestinal stromal tumor (each *n* = 1). ^b^I.e., mucosal melanoma. ^c^Including one salvage esophagectomy after primary radio-chemo-therapy and one gastrointestinal stromal tumor. ^d^Postoperative pathological “T” stage of primary adenocarcinoma and squamous cell carcinoma of the esophagus [concerning *n* = 88 statin ( −) patients or *n* = 51 psmStatin ( −) patients, respectively, and *n* = 25 statin ( +) patients], regarding the current UICC classification. ^e^Including the one salvage esophagectomy. ^f^Oligo-metastatic disease detected intraoperatively in all patients. *psm* Propensity score matched, *BMI* Body mass index, *ASA* American Society of Anesthesiologist’s classification of physical health score, *SCC* Squamous cell carcinoma

**Table 2:** Characteristics of the surgical procedure

Variable	All patients			Propensity score-matched patients		
	Statin ( −) patients (*n* = 91)	Statin ( +) patients (*n* = 26)	*p* value	psmStatin ( −) patients (*n* = 52)	psmStatin ( +) patients (*n* = 26)	*p* value
Main procedure						
COS	63^a^ (69.2%)	23 (88.5%)	0.0758	41 (78.8%)	23 (88.5%)	0.3633
LAE	28 (30.8%)	3 (11.5%)		11 (21.2%)	3 (11.5%)	
Thoracic anastomosis	88^a^ (96.7%)	20 (76.9%)	0.0037	50 (96.2%)	20 (76.9%)	0.0145
Cervical anastomosis	3 (3.3%)	6 (23.1%)		2 (3.8%)	6 (23.1%)	
Lymph node dissection	91 (100%)	26 (100%)	1	52 (100%)	26 (100%)	1
Relevant abdomino/thoracic extended procedures (additional to esophagectomy) [*n*]	Major lung resection: 1	Major lung resection: 1		Major lung resection: 0	Major lung resection: 1	
	Minor lung resection: 4	Minor lung resection: 2		Minor lung resection: 4	Minor lung resection: 2	
	Minor liver resection: 9	Minor liver resection: 2		Minor liver resection: 4	Minor liver resection: 2	
	Jejunum catheter: 3	Jejunum catheter: 1		Jejunum catheter: 2	Jejunum catheter: 1	
	Cholecystectomy: 4	Cholecystectomy: 0		Cholecystectomy: 2	Cholecystectomy: 0	
	Colon resection: 2	Colon resection: 1		Colon resection: 0	Colon resection: 1	
	Appendectomy: 2	Appendectomy: 0		Appendectomy: 1	Appendectomy: 0	
	Omentectomy: 3	Omentectomy: 1		Omentectomy: 1	Omentectomy: 1	
	Left adrenalectomy: 1	Left adrenalectomy: 0		Left adrenalectomy: 0	Left adrenalectomy: 0	
	Other minor resections: 4	Other minor resections: 2		Other minor resections: 1	Other minor resections: 2	
*n patients*	27 (29.7%)	9 (34.6%)	0.6366	13 (25.0%)	9 (34.6%)	0.4286
Duration of the thoracic part of esophagectomy procedure [min]	126 (67–423)	127.5 (45.0–228.0)^b^	0.2078	119.5 (67.0–304.0)	127.5 (45.0–228.0)^b^	0.5955
Total duration of surgery [min]	297 (177–635)	296 (191–537)	0.9556	285.5 (177.0–534.0)	296.0 (191.0–537.0)	0.7829
Blood loss [ml]	500 (50–2500)	600 (200–4800)	0.3454	505 (100–2500)	600 (200–4800)	0.4501
IO transfusion [*n* pat]	24 (26.4%)	5 (19.2%)	0.6084	16 (30.8%)	5 (19.2%)	0.4172
PO transfusion [*n* pat]	24 (26.4%)	12 (46.2%)	0.0898	16 (30.8%)	12 (46.2%)	0.2156
Peridural anesthesia	69 (75.8%)	18 (69.2%)	0.6109	35 (67.3%)	18 (69.2%)	1

Data are given in median and minimum to maximum ranges or *n* (%). ^a^Esophago-gastrectomy with colon interposition in one case. ^b^Not available retrospectively in 2 patients. *psm* Propensity score matched, *COS* Conventional open surgery (i.e., laparotomy/thoracotomy, including patients who underwent conversion from an initially intended LAE to open surgery), *LAE* hybrid minimally invasive, laparoscopically assisted esophagectomy (i.e., laparoscopy/thoracotomy), *IO* Intraoperative, *PO* Postoperative

### Outcome analysis of the unmatched patient cohort

**Fig. 1:**
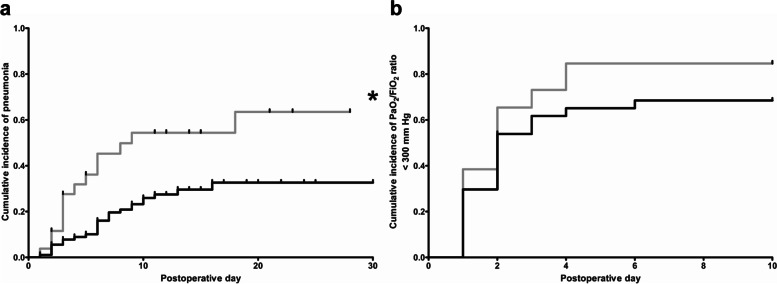
Cumulative incidences of postoperative pneumonia and postoperative reduced oxygenation index (< 300 mmHg) of the unmatched patient cohort. Black line: statin-naïve patients [statin( −) group, *n* = 91]. Gray line: patients with perioperative statin medication [statin( +) group, *n* = 26]. Patients, who were discharged, died, or suffered from re-do (revision) surgery were censored from the analysis of cumulative incidences since the day of the event. Vertical ticks indicate censored data. **a** Kaplan–Meier estimation of cumulative incidences of postoperative pneumonia of all unmatched patients. Asterisk indicates significant differences in the cumulative incidence of postoperative pneumonia between both groups at postoperative day 30 (*p* = 0.0030). **b** Kaplan–Meier estimation of cumulative incidences of reduced oxygenation index (PaO_2_/FiO_2_ < 300 mmHg) of all unmatched patients. No difference between both groups was observed at postoperative day 10 (*p* = 0.1063)

Although no differences were observed in the most relevant postoperative outcome parameters [initial extubation success, duration of mechanical ventilation or evidence for acute (re-intubation, independently from re-do surgery) or chronic (tracheotomy) respiratory insufficiency, the need for re-do surgery as well as anastomotic complications], patients from the statin( +) group had significantly higher rates of postoperative pneumonia [statin( −) patients: *n* = 30, 33.0% versus statin( +) patients: *n* = 17, 65.4%, *p* = 0.0057, OR 3.841, 95% CI 1.510–8.944]. This difference in postoperative pneumonia rates of the two groups was confirmed by the Kaplan–Meier curve comparison with log-rank test adjusted by postoperative discharge, re-do surgery, and mortality: Fig. 1a shows the difference of cumulative postoperative pneumonia incidence at POD 30 (*p* = 0.0030), whereas no differences in cumulative incidences of postoperative respiratory impairment, indicated by reduced oxygenation index (PF/R < 300 mmHg), were observed (*p* = 0.1063, Fig. 1b). In line, patients from the statin( +) group presented with significantly longer duration of postoperative stay on ICU as well as longer total duration of postoperative hospitalization (Tables 3 and 4).

**Table 3:** Postoperative C-reactive protein values and leukocyte counts in peripheral blood

Variable	All patients					Propensity score-matched patients				
	Statin ( −) patients (*n* = 91)		Statin ( +) patients (*n* = 26)		*p* value	psmStatin ( −) patients (*n* = 52)		psmStatin ( +) patients (*n* = 26)		*p* value
Leukocytes [giga/l]		MV		MV			MV		MV	
POD 0 (on arrival at ICU)	9.2 (3.6–29.6)	1	8.9 (3.0–24.6)	0	0.5736	9.5 (3.7–29.6)	1	8.9 (3.0–24.6)	0	0.5180
POD 1	10.4 (4.0–23–6)	1	10.7 (5.3–23.1)	0	0.4664	10.2 (4.4–23.6)	0	10.7 (5.3–23.1)	0	0.5076
POD 2	11.2 (1.8–24.6)	0	11.5 (6.7–19.0)	0	0.4607	10.4 (1.8–24.6)	0	11.5 (6.7–19.0)	0	0.4297
POD 3	8.9 (4.4–34.1)	8	9.5 (1.9–17.9)	0	0.9010	8.9 (4.4–34.1)	2	9.5 (1.9–17.9)	0	0.8652
POD 4	7.6 (3.9–106.0)	14	8.4 (2.7–18.2)	1	0.5285	7.4 (3.9–106.0)	7	8.4 (2.7–18.2)	1	0.5728
POD 5	7.7 (3.4–21.5)	23	8.4 (3.9–16.7)	2	0.5572	7.7 (3.4–16.6)	13	8.4 (3.9–16.7)	2	0.7989
POD 6	8.7 (3.4–23.1)	26	9.9 (5.8–15.2)	5	0.2070	9.3 (3.4–19.2)	12	9.9 (5.8–15.2)	5	0.5286
POD 7	9.4 (3.0–29.0)	25	10.7 (5.4–15.8)	5	0.2578	9.8 (3.0–29.0)	16	10.7 (5.4–15.8)	5	0.4923
POD 8	11.0 (4.1–33.3)	36	11.0 (5.9–22.4)	5	0.8800	10.7 (4.1–31.5)	17	11.0 (5.9–22.4)	5	0.8990
POD 9	11.6 (4.5–49.7)	30	11.5 (6.2–22.3)	3	0.8923	12.3 (4.9–40.7)	17	11.5 (6.2–22.3)	3	0.8115
POD 10	13.0 (4.2–38.7)	37	11.3 (7.1–28.2)	5	0.7101	12.4 (4.7–37.8)	18	11.3 (7.1–28.2)	5	0.7950
C-reactive protein [mg/l]		MV		MV			MV		MV	
POD 0 (on arrival at ICU)	5.2 (0–256.0)	1	5.2 (0.5–106.7)	2	0.7730	8.7 (0.0–256.0)	0	5.2 (0.5–106.7)	2	0.6670
POD 1	93.4 (31.2–226.2)	1	88.4 (48.9–155.3)	0	0.8296	94.4 (35.4–226.2)	0	88.4 (48.9–155.3)	0	0.7385
POD 2	202.8 (55.3–359.4)	0	211.0 (110.7–329.3)	0	0.2472	194.0 (55.3–359.4)	0	211.0 (110.7–329.3)	0	0.2091
POD 3	185.1 (68.5–365.4)	8	234.1 (26.3–403.9)	0	0.1290	186.3 (68.5–365.4)	2	234.1 (26.3–403.9)	0	0.0683
POD 4	156.9 (30.1–405.8)	14	194.3 (85.8–410.0)	1	0.0557	156.9 (51.4–405.8)	7	194.3 (85.8–410.0)	1	0.0931
POD 5	138.1 (25.4–348.0)	23	150.1 (40.7–539.1)	2	0.4632	139.9 (25.4–345.2)	13	150.1 (40.7–539.1)	2	0.4748
POD 6	124.5 (6.0–423.2)	25	148.8 (36.8–390.9)	5	0.3188	123.8 (14.1–423.2)	12	148.8 (36.8–390.9)	5	0.3351
POD 7	122.5 (8.3–445.1)	25	135.4 (19.9–316.4)	5	0.4019	133.7 (8.3–445.1)	16	135.4 (19.9–316.4)	5	0.6611
POD 8	147.1 (6.0–431.8)	36	213.6 (12.0–491.9)	5	0.4714	147.1 (6.0–431.8)	17	213.6 (12.0–491.9)	5	0.6116
POD 9	138.2 (5.6–412.9)	32	153.2 (22.1–446.9)	3	0.3319	138.2 (5.6–412.9)	17	153.2 (22.1–446.9)	3	0.4550
POD 10	148.7 (4.9–393.9)	37	159.8 (22.9–310.5)	5	0.3018	127.2 (8.1–393.9)	18	159.8 (22.9–310.5)	5	0.2638

Data are given in median and minimum to maximum ranges. *MV* Missing values, including patients who died during postoperative day 0–10, *psm* Propensity score matched, *POD* Postoperative day

**Table 4:** Postoperative outcome

Variable	All patients			Propensity score-matched patients		
	Statin ( −) patients (*n* = 91)	Statin ( +) patients (*n* = 26)	*p* value	psmStatin ( −) patients (*n* = 52)	psmStatin ( +) patients (n = 26)	*p* value
PO hospital stay						
Total [d]^a^	17 (9–141)	25 (11–75)	0.0068	16.5 (9–141)	25 (11–75)	0.0185
Initial PO stay on ICU [d]^a^	4 (1–76)	6 (2–58)	0.0729	5 (1–76)	6 (2–58)	0.2556
Return to ICU [n patients]	17 (18.7%)	3 (11.5%)	0.5578	8 (15.4%)	3 (11.5%)	0.7429
Cumulative PO stay on ICU [d]^a^	5 (1–84)	11.5 (2–58)	0.0221	5 (1–84)	11.5 (2–58)	0.0612
Initial postoperative extubation [h]	0 (0–11)	0 (0–4)	0.8171	0 (0–11)	0 (0–4)	0.8666
Cumulative perioperative mechanical ventilation [h]	12.6 (4.8–2280)	13.5 (5.2–788.3)	0.5184	12.7 (5.3–876.9)	13.5 (5.2–788.3)	0.6146
Pneumonia^b^	30 (33.0%)	17 (65.4%)	0.0057	20 (38.5%)	17 (65.4%)	0.0317
Pneumonia diagnosis on POD	6 (1–25)	4 (1–18)	0.1390	6 (1–25)	4 (1–18)	0.2507
Tracheotomy	13 (14.3%)	7 (26.9%)	0.1462	10 (19.2%)	7 (26.9%)	0.5619
Extubation in the operating room	22 (24.2%)	6 (23.1%)	1	12 (23.1%)	6 (23.1%)	1
Initial extubation during the first 12 h postoperatively	75 (82.4%)	21 (80.8%)	0.7808	42 (80.8%)	21 (80.8%)	1
Re-intubation^c^	22 (24.2%)	10 (38.5%)	0.2111	15 (28.9%)	10 (38.5%)	0.4453
Perioperative oxygenation index < 300 mm Hg [*n* patients]^d^						
First intraoperative	21 (23.1%)	6 (23.1%)	1	14 (26.9%)	6	0.7891
POD 0 (on arrival at ICU)	32 (35.2%)	8 (30.8%)	0.3429	22 (42.3%)	8	0.4594
POD 1	27 (29.7%)	10 (38.5%)	0.4743	19 (36.5%)	10	1
POD 2	37 (40.7%)	14 (53.9%)	0.2667	20 (38.5%)	14	0.2312
POD 3	28 (30.8%)	14 (53.9%)	0.0382	17 (32.7%)	14	0.0889
POD 4	19^*^ (21.1%)	12 (46.2%)	0.0215	10^*^ (19.6%)	12	0.0187
POD 5	17^*^ (18.9%)	10 (38.5%)	0.0624	11^*^ (21.6%)	10	0.1752
POD 6	21^*^ (23.3%)	7^*^ (28.0%)	0.6087	12^*^ (23.5%)	7^*^	0.7795
POD 7	19^*^ (21.1%)	10^*^ (40.0%)	0.0695	14^*^ (27.5%)	10^*^	0.3015
POD 8	20^*^ (22.2%)	10^*^ (40.0%)	0.1201	12^*^ (23.5%)	10^*^	0.1799
POD 9	19^*^ (21.1%)	7^*^ (28.0%)	0.5890	12^*^ (23.5%)	7^*^	0.7795
POD 10	17^*^ (18.9%)	7^**^ (29.2%)	0.2730	10^*^ (19.6%)	7^**^	0.3860
Re-do (revision) surgery during POD 1–30	12 (13.2%)	9 (34.6%)	0.2278	10 (19.2%)	6 (34.6%)	0.7690
Anastomotic complications^e^	18 (19.8%)	6 (23.1%)	0.7842	11 (21.2%)	6 (23.1%)	1
PO 30-day mortality [*n*]	6 (6.6%)	3 (11.5%)	0.4139	5 (9.6%)	3 (11.5%)	1

Data are given in median and minimum to maximum ranges or *n* (%). ^a^Patients who suffered from in-hospital mortality were excluded from the analysis of postoperative length of stays. ^b^Overall pneumonia rate, irrespectively from re-do surgery. ^c^Re-intubation due to acute respiratory insufficiency excluding re-intubations due to re-do (revision) surgery. ^d^Irrespectively from re-do surgery; patients who died during POD 0–10 were excluded from the analysis upon their death: asterisks (* and **) indicate the number of deaths on the respective postoperative day. ^e^Anastomotic complications, including insufficiency and/or gastric tube necrosis requiring therapy (stent, endo-vacuum therapy, or re-do surgery). *psm* Propensity score matched; *PO* Postoperative, *ICU* Intensive care unit, includes medium care unit; *POD* Postoperative day

### Outcome analysis after propensity score matching

**Fig. 2:**
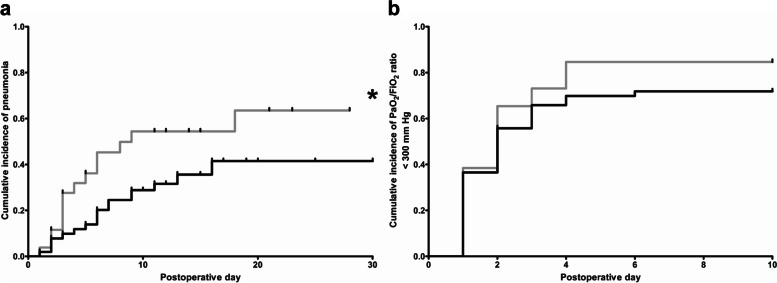
Cumulative incidences of postoperative pneumonia and postoperative reduced oxygenation index (< 300 mmHg) of the matched patient cohort by propensity score. Black line: statin-naïve patients [statin( −) group, *n* = 52]. Gray line: patients with perioperative statin medication [statin( +) group, *n* = 26]. Patients who were discharged, died, or suffered from re-do (revision) surgery were censored from the analysis of cumulative incidences since the day of the event. Vertical ticks indicate censored data. **a** Kaplan–Meier estimation of cumulative incidences of postoperative pneumonia of matched patients. Asterisk indicates significant differences in the cumulative incidence of postoperative pneumonia between both groups at postoperative day 30 (*p* = 0.0468). **b** Kaplan–Meier estimation of cumulative incidences of reduced oxygenation index (PaO_2_/FiO_2_ < 300 mmHg) of matched patients. No difference between both groups was observed at postoperative day 10 (*p* = 0.2980)

The 26 patients from the statin( +) group were matched with 52 statin-naïve patients by propensity score, which was calculated by relevant variables of preoperative patient or procedure characteristics. Thereafter, relevant characteristics of the study groups were widely balanced (Tables 1 and 2). The main results of the native two-group comparison were confirmed by the analysis of the matched patient cohorts. Even after PSM, postoperative pulmonary outcome was significantly impaired in patients from the statin( +) group reflected by both a significantly higher postoperative pneumonia rate in the two-group comparison (*p* = 0.0317, OR 3.022, 95% CI 1.165–7.892, Tables 3 and 4) as well as a higher cumulative pneumonia incidence calculated by Kaplan–Meier estimation and log-rank test (*p* = 0.0468, Fig. 2a), whereas still no differences in cumulative incidences of postoperative respiratory impairment, indicated by reduced oxygenation index (PF/R < 300 mmHg), were observed (*p* = 0.2980, Fig. 2b).

### Correlations between statin medication, postoperative pneumonia, and markers of inflammation

Perioperative statin medication correlated significantly with the pneumonia rate of both the unmatched (*r*_sp_ = 0.275, *p* = 0.0027) as well as propensity score-matched patient cohorts (*r*_sp_ = 0.254, *p* = 0.0247, Table 5). Although no obvious differences were observed between the unmatched and matched patient groups regarding perioperative markers of inflammation, pneumonia vice versa results in positive correlations with postoperative CRP values and leukocyte counts beyond postoperative days 3 or 4, respectively (Tables 4 and 5). However, this effect was attenuated in statin( +) patients (Table 5).

**Table 5:**
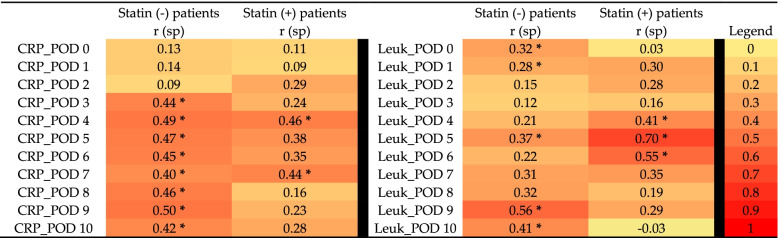
Correlation analysis

Values of the propensity score-matched patient cohorts were included in Spearman’s rho rank correlation analysis (i.e., *n* = 52 statin-naïve and *n* = 26 patients, who received statin medication perioperatively). Correlations between postoperative pneumonia and postoperative C-reactive protein (CRP) values as well as postoperative leukocyte (Leuk) counts in peripheral blood immediately after surgery (POD0) and during postoperative days (POD) 1–10 are presented by the heatmap with no correlation (*r*_sp_ = 0) in yellow to strong correlation (*r*_sp_ = 1) in red. Asterisks indicate statistical significance (*p* value ≤ 0.05)

## Discussion

### Postoperative pulmonary outcome

Patients after abdomino-thoracic esophagectomy are highly vulnerable for the development of postoperative respiratory impairment as well as pulmonary complications (Reichert et al. 2020, 2019). Development of PPC after abdomino-thoracic esophagectomy is caused by a multifactorial pathogenesis and impairs patient outcome dramatically (Molena et al. 2014); hence, investigation and modulation of factors contributing to postoperative pneumonia are mandatory to sustainably improve perioperative patient care after this type of high-risk surgery. The results of the present work are in line with the hypothesis, whether statin medication has an influence on postoperative pulmonary outcome concerning pneumonia development in patients after abdomino-thoracic esophagectomy. These effects were shown in unmatched patient cohorts and were constantly confirmed after PSM of statin-naïve patients with the statin( +) patient cohort. Thereby, patients under perioperative statin medication were investigated with an impaired pulmonary outcome by increasing pneumonia rates after abdomino-thoracic esophagectomy. This harmful effect in statin( +) patients was shown in the absence of other factors, which had been previously evaluated as possible contributors to pulmonary morbidity, including rates of conventional open or hybrid minimally invasive, laparoscopically assisted surgery, the duration of the abdominal part of abdomino-thoracic esophagectomy or preoperative induction (chemo-irradiation-) therapy (Reichert et al. 2020, 2019; Mariette et al. 2019; Schroder et al. 2019; Reynolds et al. 2006).

### Potential effects of statin medication on the development of postoperative pneumonia

Concerning initial dysbalances in patient characteristics between both the statin( −) and statin( +) patient cohorts, one has to be aware that statin medication is indicated for the primary as well as secondary prophylaxis of thromboembolic cardiovascular events in lipidemic and atherosclerotic patients. This might be the reason why patients from the statin( +) group were older and suffered more frequently from chronical illness and it must be noted that the slightly sicker patients were filtered out of the group of statin-naïve patients by propensity score for comparison. Whether the statin effect on pulmonary outcome after esophagectomy might be also evident in younger and more healthy patients at the time of surgery remains elusive from the present data analysis. Apart from the lipid-lowering effect of statins through the competitive inhibition of 3-hydroxy-3-methyl-glutaryl-CoA reductase (Lemos et al. 2004), these drugs have some properties that interfere with inflammatory responses. Statins suppress the expression of adhesion molecules and consecutively inhibit the activation and recruitment of inflammatory cells and decrease inflammasome activity as well as production of pro-inflammatory cytokines including Il-1, Il-6, TNF-α, and CRP in vitro as well as in vivo (Koushki et al. 2020; Meng et al. 2020). Thereby, some of the anti-inflammatory effects of statins are mainly responsible for plaque stabilization in atherosclerotic diseases (Koushki et al. 2020), but statins also systemically affect innate immunity and host defense (Koushki et al. 2020; Meyer et al. 2020; Meng et al. 2020; Jesus Oliveira et al. 2020; Oliveira et al. 2020). The role of statins in the regulation of inflammasome activation, especially NLRP3 and consecutively the interleukin-1 release, remains controversial; however, statins suppress Toll-like receptor 4 signaling and NF-*k*B pathway, which are key players in innate immunity (Koushki et al. 2020; Meyer et al. 2020). In line with these findings, atorvastatin affects peripheral blood mononuclear cells with reduced production of pro-inflammatory cytokines, including TNF-*a* and interleukin-6 (Oliveira et al. 2020). Nevertheless, the actions of statins on the immune system are complex and multifaceted and by now their anti-inflammatory effects and their role in innate immunity are not completely understood (Koushki et al. 2020). Especially the impact of statin medication on perioperative inflammation and postoperative complication development has been poorly investigated until now. Notably, in the present study, the most commonly used statins were lipophilic including simvastatin and atorvastatin (23 of 26 patients). Slight differences in their pleiotropic effects had been reported in the literature, but even hydrophilic statins as pravastatin [used in the remaining three statin( +) patients] have similar effects on inflammatory pathways (Koushki et al. 2020). In the past, Krivoy et al. showed a reduction of systemic CRP levels during high-dosage therapy with atorvastatin after coronary artery bypass graft surgery (Krivoy et al. 2008). However, the statin dosages within the therapeutic ranges applied in patients of the present study were much lower compared with those being effective on postoperative systemic CRP levels in the experimental setting reported by Krivoy and colleagues (Krivoy et al. 2008). But the findings with a suppressed CRP response upon inflammatory stimuli go in line with correlation analyses from our study. In correlation analyses, we found a rather strong and significant correlation between development of postoperative pneumonia, an infectious complication, which is regularly diagnosed during the early postoperative phase (Reichert et al. 2020, 2019), and postoperative CRP values in statin( −) patients. This CRP response corresponding to pneumonia development was attenuated in statin( +) patients, although the rate of postoperative pneumonia correlated significantly with statin medication. The latter might be a detrimental result of an impaired host defense in patients under statin medication driven by the interruption of various pro-inflammatory pathways. Also, Meyer et al. recently described the impact of statin medication on perioperative systemic inflammatory reaction after lung cancer surgery (Meyer et al. 2020). Although they found no impact of perioperative statin medication on postoperative markers of systemic inflammation in their overall results, they investigated a gender-specific effect of statin-naïve and statin-taking patients on postoperative systemic CRP levels (Meyer et al. 2020). Furthermore, they confirmed their clinical results by a translational research approach and pronounced that statin medication the other way around leads to a more pro-inflammatory phenotype upon (surgical) trauma on a monocytic level in their experimental setting (Meyer et al. 2020).

These immunomodulatory actions of statins might be beneficial in diseases caused by chronic inflammation or autoimmunity, e.g., rheumatoid arthritis and multiple sclerosis, or an overwhelming and misdirected immune reaction, e.g., in the early phase of sepsis (Jesus Oliveira et al. 2020; Patel et al. 2012) as well as allograft rejection after transplantation (Johnson et al. 2003). However, immunomodulation to an anti-inflammatory phenotype might be harmful and impairs host defense under infectious conditions. The results of the present study show significantly higher rates of postoperative pneumonia in patients under statin medication after esophagectomy, a surgical procedure which results in an extended surgical trauma to the chest and a highly vulnerable lung (Reichert et al. 2020, 2019). Although no differences were observed in the postoperative systemic inflammatory response in the reported patient cohorts, indicated by systemic CRP levels, the extraordinarily high rate of postoperative pneumonia development in statin( +) compared with statin-naïve patients after abdomino-thoracic esophagectomy might be a result of the anti-inflammatory or — in cases of impaired host defense against bacterial infection by suppression of TLR4 pathways — immunosuppressive effects through perioperative statin medication. Impaired systemic host defense against bacterial infection of the lung as well as the inadequate reaction of local, pulmonary monocytes upon surgical trauma-induced CRP as interpreted from the work by Meyer et al. (Meyer et al. 2020) may result in high pneumonia rates, impaired oxygenation on postoperative days 3 and 4, impaired systemic inflammatory response to pneumonia investigated in the correlation analyses, prolonged postoperative critical illness, and prolonged length of postoperative stay at the intensive care unit as well as longer duration of total hospitalization in statin( +) patients.

By contrast, some authors reported beneficial effects of statin use in patients who underwent non-cardiac surgery. Recently, Komatsu and colleagues published a data bank analysis and reported slightly reduced rates of postoperative respiratory complications, reduced in-hospital mortality, and shorter duration of hospitalization in statin users after non-cardiac surgery (Komatsu et al. 2020). Also, London and Berwanger and their colleagues found a reduction of risk for complications, especially of cardiovascular morbidity in patients under perioperative statin medication from their large propensity score-matched data bank analyses of non-cardiac surgery (London et al. 2017; Berwanger et al. 2016), as well as in the meta-analysis by Ma et al., the reduction of postoperative cardiovascular complications was confirmed for statin users after non-cardiac surgery (Ma et al. 2018). However, in the prospectively randomized controlled LOAD trial, these beneficial cardiovascular effects were not reproduced under medication with atorvastatin (Berwanger et al. 2017). Nevertheless, in these studies, major thoracic surgery, especially abdomino-thoracic esophagectomy is highly underrepresented (Berwanger et al. 2016, 2017); thus, a generalization and transfer of the results to perioperative patient care and complication rates after esophagectomies might not be suitable. In a prospectively randomized controlled trial including 31 patients by Shyamsundar et al., perioperative high-dosage treatment with simvastatin compared with placebo resulted in deceased levels of systemic inflammatory markers, but did not influence on the rate of PPC (Shyamsundar et al. 2014).

Nevertheless, as abdomino-thoracic esophagectomies are surgical procedures with an extraordinarily high risk for severe, especially pulmonary complications, the results of the present study should be noted with utmost care. In a previous work, we have shown high rates of impairments in pulmonary function measured by reduced P/F ratios after abdomino-thoracic esophagectomy and compared them with other types of major thoracic surgery, even those including major trauma to the chest cavity and single-lung ventilation during surgery (Reichert et al. 2019). Herein, we investigated dramatic impairments of lung physiology and pulmonary function (objectified by P/FR perioperatively) after esophagectomy (Reichert et al. 2019). Therefore, the rate of early impairment of pulmonary function after esophagectomy is not mainly influenced by the extent of the trauma and even pain caused by the laparotomy during the abdominal part nor injury of the thoracic cavity and the lung during the thoracic part of the esophagectomy procedure (Reichert et al. 2020). Nevertheless, the incidence of postoperative pneumonia beyond POD 3, which seems to be the day with the highest vulnerability of the lung and consecutively highest risk for developing lung injury following esophagectomy, might be associated with the surgical trauma-induced release of danger-associated molecular patterns (DAMP) which then indirectly harm the lung and consecutively increase the risk to acquire pneumonia (Reichert et al. 2020; D’Journo et al. 2010; Okamura et al. 2015; Katsuta et al. 1998). Thus, a more severe initial DAMP-induced systemic inflammation might have impaired host defense against pneumonia-associated pathogens (Reichert et al. 2020; Babic et al. 2020). We speculate that alterations of local inflammatory response in the lung against pneumonia-associated pathogens upon statin medication lead to a local anti-inflammatory or more specifically immunosuppressive phenotype which then impairs local host defense. This harmful statin effect would result in the higher rate of postoperative pneumonia with a consecutively impaired postoperative patient outcome and prolonged postoperative hospitalization in statin( +) patients of the present study.

### Causal role of statins in the development of postoperative pneumonia

According to Bradford Hill’s criteria (Hill 1965), the present work investigates a causal role of perioperative statin medication in the development of postoperative pneumonia after abdomino-thoracic esophagectomy. The strength effect size of perioperative statin medication on postoperative pneumonia development was demonstrated by the OR 3.022 in the analysis of the PSM-matched patient cohorts. Thereby, the significant differences in postoperative pneumonia between both the highly specific statin( −) and statin( +) patient cohorts were consistently observed by the different statistical methods performed. As coherently discussed above, the literature provides plausible explanations of the extent to which statins influence inflammatory pathways and thus favor the development of postoperative pneumonia after esophagectomy. However, further experimental and clinical studies are necessary to investigate confounding factors in patient characteristics, different types of statins, and dose–response relationships, which might influence the effect size of perioperative statin medication on postoperative pneuomonia development.

### Limitations of the present study and future aspects

Although relevant patient characteristics, which might influence on postoperative pulmonary outcome after abdomino-thoracic esophagectomy including chronic cardiovascular and pulmonal diseases, were balanced between both groups after PSM, the retrospective character with a relatively small sample size is the strongest limitation of the present study. Hence, no firm conclusions should be drawn from the results, but the present study allows the creation of testable hypotheses for larger-scaled retrospective patient data analyses or prospectively conducted trials regarding the effects of perioperative statin medication to develop strategies that make esophageal surgery safer for our patients.

## Conclusion

In conclusion, the pathogenesis of pulmonary morbidity in response to esophagectomy is complex and our results suggest that statin medication might contribute to postoperative pneumonia development. Because the main indication for statin medication is prophylaxis of cardiovascular events for atherosclerotic patients in the long term and although some retrospective studies have shown beneficial effects of statin medication on cardiovascular morbidity after non-cardiac surgery, the results of the present study might support the conclusion to interrupt statin medication perioperatively in appropriate patients with a low risk for severe cardiovascular morbidity undergoing esophagectomy and to prevent them from exceptionally high rates of postoperative pulmonary morbidity. Therefore, this hypothesis generated from our retrospective study needs to be further investigated by prospectively conducted trials.

## Data Availability

The datasets used and/or analyzed during the current study are available from the corresponding author on reasonable request.

## Abbreviations

ASA: Anesthesiologist’s classification of physical health score
CCI: Charlson Comorbidity Index
CRP: C-reactive protein
FiO_2_: Fraction of inspired oxygen
ICU: Intensive care unit
Il: Interleukin (Il)
NF-*k*B: Nuclear factor *k*B
P/FR: Oxygenation index (i.e., PaO_2_/FiO_2_ ratio)
PaO_2_: Arterial pressure of oxygen
POD: Postoperative day
PPC: Postoperative pulmonary complications
PSM: Propensity score pair matching
*r*_sp_: Spearman’s rank correlation coefficient
TNF-α: Tumor necrosis factor-α
